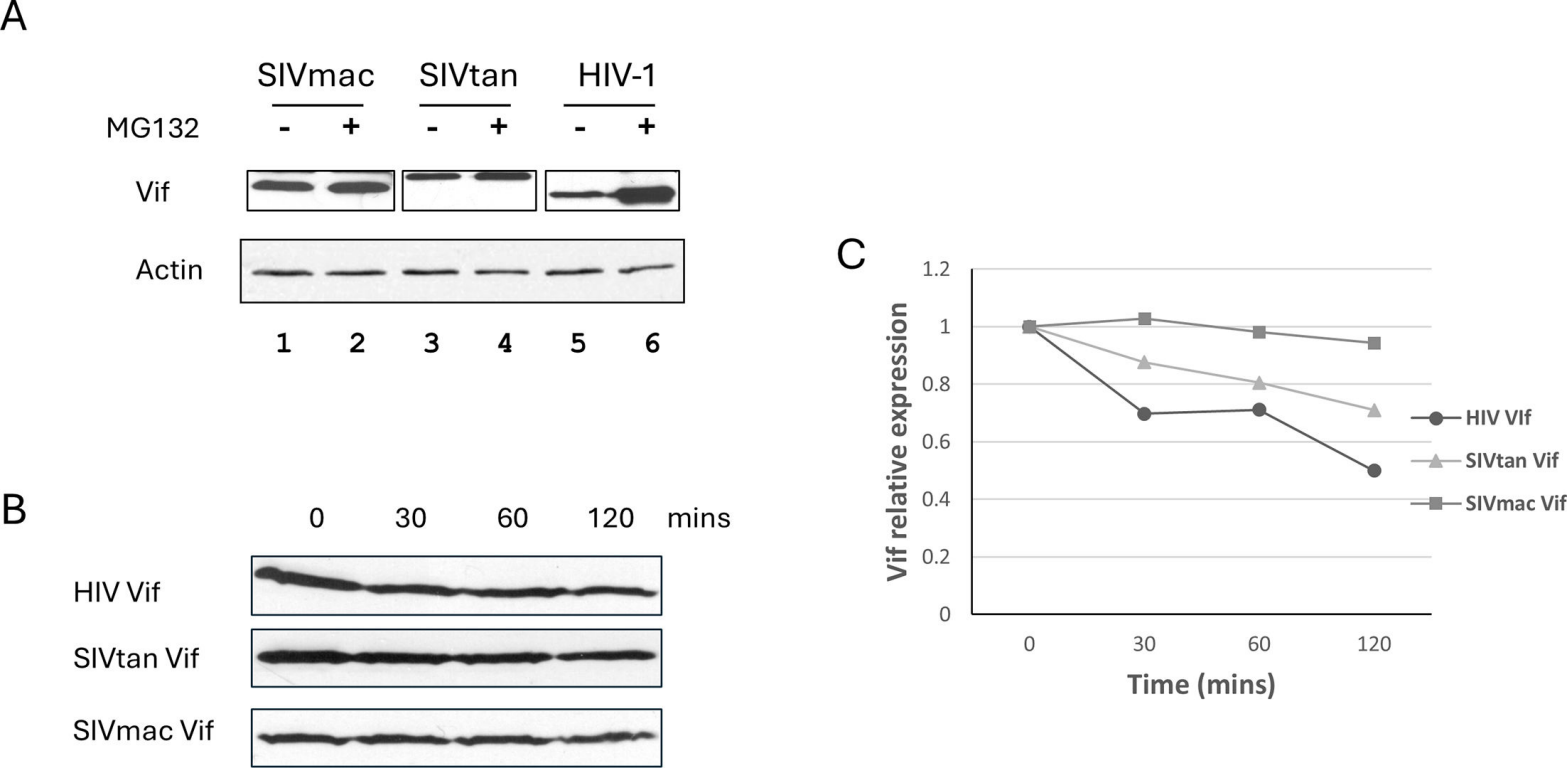# Correction for Shao et al., “Polyubiquitination of APOBEC3G Is Essential for Its Degradation by HIV-1 Vif”

**DOI:** 10.1128/jvi.01368-25

**Published:** 2025-09-23

**Authors:** Qiujia Shao, Yudi Wang, James E. K. Hildreth, Bindong Liu

## AUTHOR CORRECTION

Volume 84, no. 9, p. 4840–4844, 2010, https://doi.org/10.1128/jvi.01911-09. Page 4841: Figure 1A, B, and C should appear as shown in this correction. During figure assembly, the Western blot image for SIVtan Vif was inadvertently duplicated and also placed in the SIVmac Vif panel (panel B). This unintentional oversight has been corrected by inserting the correct SIVmac Vif image, and the densitometry analysis (panel C) has been revised accordingly. We also clarified panel A by adding a dividing line between the different Vifs to prevent any confusion.

We sincerely regret this oversight. These clerical errors do not impact the data interpretation or the conclusions of the article.

**Fig 1:**